# The policy implementation gap of school oral health programmes in Tshwane, South Africa: a qualitative case study

**DOI:** 10.1186/s12913-020-05122-8

**Published:** 2020-04-21

**Authors:** Mpho Molete, Aimee Stewart, Edna Bosire, Jude Igumbor

**Affiliations:** 1 Department of Community Dentistry, University of the Witwatersrand, School of Oral Health Sciences, 7 York Road, Parktown, Johannesburg, 2193 South Africa; 2 University of the Witwatersrand, School of Therapeutic Sciences, Johannesburg, South Africa; 3 School of Cinical Medicine, University of the Witwatersrand, Development Pathways for Health Research Unit (DPHRU), Johannesburg, South Africa; 4 University of the Witwatersrand, School of Public Health, Johannesburg, South Africa

**Keywords:** Health policy, Implementation, School health, Oral health

## Abstract

**Background:**

School going children across the world continue to experience high levels of untreated dental diseases. The South African Oral Health policy documents present measures to address the oral health needs of children in school settings, yet the burden of oral disease in the country is over 50% among primary school children.

**Methods:**

Our study therefore sought to assess the implementation of school oral health programmes in Tshwane in line with policy recommendations using the Walt & Gilson policy analysis triangle. A qualitative explanatory case study was undertaken using a combination of data from direct observations and interviews. The case analysis involved assessing the processes of providing school oral health programmes that were offered at 10 schools in Tshwane. The measuring tools included process maps and an interview guide.

**Results:**

The results found that policy implementation was affected by poor prior planning, inadequate resources, poor school infrastructure and lack of support from key stakeholders. Furthermore, inconsistencies in policy interpretation by management, coupled with the fact that the oral hygienists were not conversant with the policy hampered delivery of the policy content. The variations in policy implementation observed were often at the discretion of the oral hygienist in response to contextual challenges.

**Conclusion:**

There was policy and practice misalignment and variations in the processes of implementing oral health programmes across the 10 schools. Hence regular monitoring, evaluation and root cause analysis is recommended for such programmes in order to make informed decisions on contextually relevant and standardised programme modifications.

## Background

Most oral health conditions are preventable and their early onset is reversible [1, 2]. Yet, school going children across the world continue to experience high levels of untreated dental diseases [3, 4]. South African Oral Health policy presents measures to address the oral health needs of children in school settings [5]. Additionally, schools provide an ideal opportunity to reach children with oral health education, skills and other differentiated services [2, 6, 7]. The South African public school system is also an opportune vehicle to address oral health needs of children with its over 88% enrolment of children aged 5 years and older [8, 9]. In addition, the inextricable link between oral health of children and schools is demonstrated in the association between poor oral health status and poor school performance [10, 11].

School oral health programmes have existed for many years in South Africa but the burden of oral health disease persisted between 2002 and 2015 with the prevalence of dental caries among 6 year olds ranging between 46 and 73% [12–14]. South Africa’s school oral health policies seek to respond to local oral health needs while learning from global best practices [2]. Hence there are existing policies such as the South African National Oral Health Strategy (2010), the Integrated School Health Policy document (2012); and the School Health Policy and Implementation Guidelines (2011).

These policies recommend that school oral health programme deployment processes should include appropriate community and resource assessments before school selection and entry. Other key steps include the mass mobilization of school staff, caregivers and pupils in order to obtain acceptance and co-operation for programme delivery. Oral health needs of pupils are to be identified and targeted services offered to specific age groups. The targeted services should include oral health screening, fissure sealant placement on permanent molar teeth, fluoride varnish applications and provision of Atraumatic Restorative Technique (ART). In addition to the services mentioned, the oral hygienists are expected to introduce and sustain preventive activities such as the tooth brushing programme.

The proposed processes of implementation are emphasized differently in each of the main South African documents with essential information concerning the implementation of the services being disjointed across the three policy documents. In addition, there are no clear guidelines of how to carry out the specific recommended tasks [2, 15, 16]. It therefore becomes apparent that policy inconsistencies may potentially conflate oral health service delivery efforts [17].

Primary health care (PHC) revitalization is central to ongoing health reforms in South Africa as this is a platform for service delivery, health promotion facilitation, prevention of illness and a first point of entry into the healthcare sector [18]. It comprises of three complementary streams which are currently being piloted at 11 provincial sites across the country. Since 2011 the District of Tshwane located in the Gauteng province of South Africa has been one of the pilot sites for school health services. The schools in the district are categorized into quintiles based on their socio-economic status. Quintile 1 schools are located in the poorest areas while quintile 5 schools are in higher socio-economic areas [19]. Oral health services are included in the quintile 1 and 2 school health services.

Our study therefore sought to assess the processes of implementation of school oral health programmes in Tshwane against policy recommendations. Given the complexities of policy implementation, the Walt & Gilson triangle was used to assess the key elements at play within the implementation process [20]. The triangle highlights how the elements of policy content, processes and context influence the extent of policy implementation and emphasizes the central role of actors in the decision making processes and their influence on the three elements [21, 22]. The Walt & Gilson triangle was therefore used to guide our analysis and to delineate key elements in the current school oral health programmes for clearer benchmarking against policy recommendations.

## Methods

A qualitative explanatory case study was undertaken to clarify the implementation processes of school oral health programmes in Tshwane. The study was conducted between 2017 and 2018 using a combination of data from observations and interviews [23]. There were 10 oral hygienists providing oral health services at various schools in the district, each one being responsible for approximately four schools in a year, hence the 10 oral hygienists were purposively sampled to participate in our study as they managed the programmes at the schools. One school from each of the oral hygienist’s list of schools that have been operational for more than 1 year was selected to participate in the study.

### Data collection and tools

The data collection tools namely the process maps and interview guides were informed by using the prescripts and recommendations of provincial and national oral health policy documents and the WHO school oral health guidelines [2, 5], see Additional file 1. Process maps were used to guide the observations as the oral hygienists carried out their activities at the chosen schools. These maps represented a visual illustration of school oral health service delivery components and were used as an analytical tool to delineate and document the sequence of activities and events followed by the hygienist at each school [24]. Furthermore the mapping process assisted in understanding the interactive nature of the policy implementation process. Semi-structured face to face interviews were then conducted with the hygienists upon completion of the observations to explore the reasons for the steps followed and services offered.

**Fig. 1:**
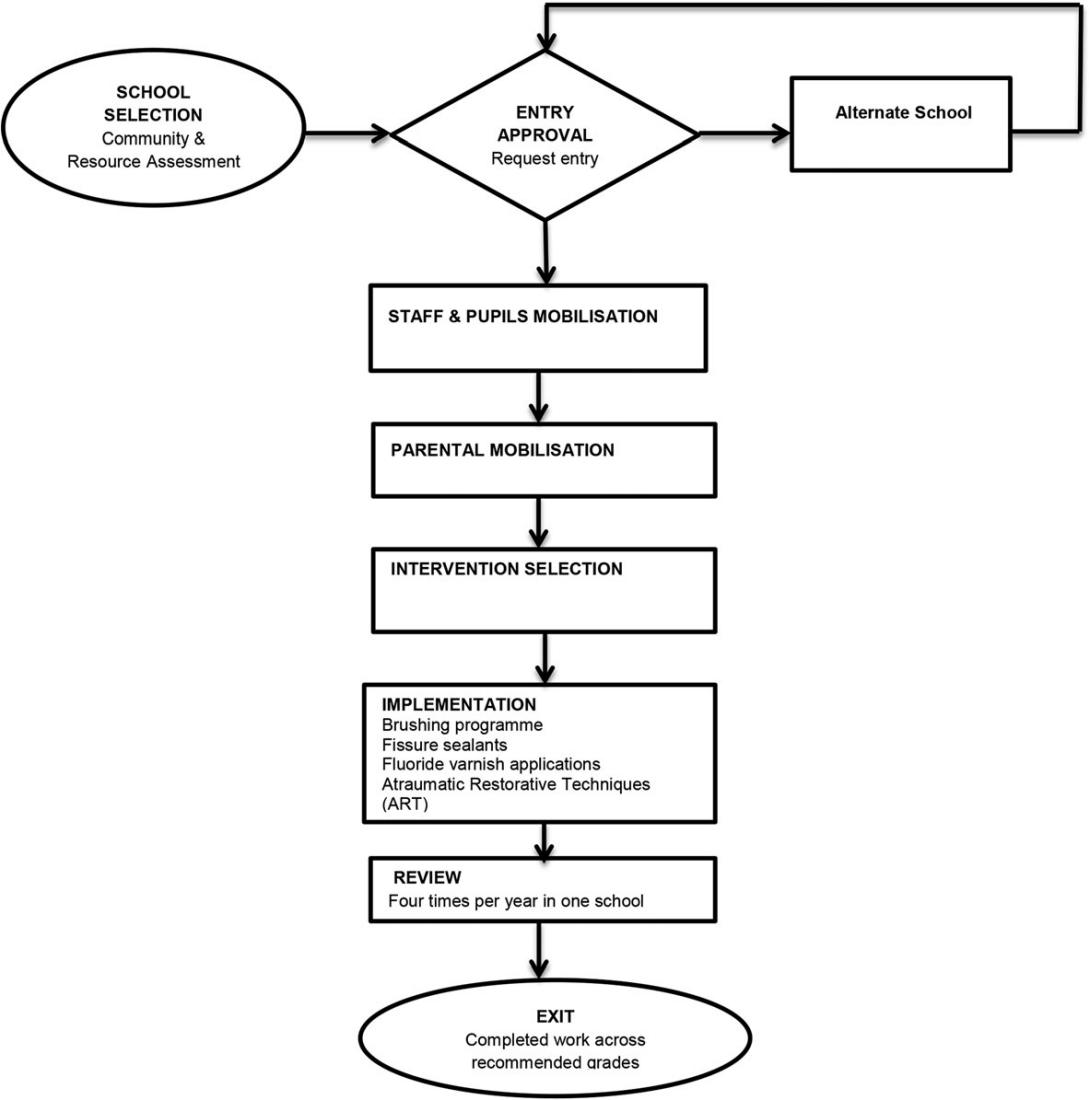
Map of proposed implementation. This map was compiled using information from the South African National Oral Health Strategy and the Integrated School Health Policy document [5, 15]

The observations were carried out as each of the oral hygienists was performing their activities at the various schools. Observations took place during one of their morning sessions, lasting approximately 4 hours at a particular school. The stepwise process illustrated in Fig. 1 was used to benchmark actual observations at the schools for compliance. Findings from the observations facilitated more probing and exploring of contextual barriers and drivers to the incorporation of oral health at the schools.

When the hygienists completed their procedures, individual interviews were conducted with the purpose of gaining an understanding of why implementation was undertaken in a particular way. The first author encouraged the oral hygienists to provide information on the actual processes that they followed during programme implementation, as opposed to stating the ideal process as outlined in the policy documents [25]. Interviews lasted between 30 and 45 min and were conducted in a private space within the school. Detailed field notes describing the school contexts, activities, and processes were also taken during data collection; these were examined by the other authors in order to ensure credibility of the data.

### Data analysis

A thematic framework analyses was used for analysing the data, this involved the key processes of; Familiarisation; Identifying a thematic framework; Indexing; Charting; Mapping and Interpretation [26]. The transcripts were read repeatedly for familiarisation of the data by the first and third authors, this permitted for identification of ideas, making observations, and getting insights and inferences. Upon familiarisation of the data, initial codes were derived from the Walt & Gilson triangle in order to guide the development of a thematic framework that was used for identifying the rest of the codes inductively. Coding was conducted until no further codes were identified and the data were considered to be saturated. Indexing and charting of the data involved merging of the codes into patterns of similarities and differences and aligning them to particular themes and sub-themes. The data were then integrated as we assessed the links among the data and brought about an understanding of events, processes and context. In addition, interpretation of the data was linked back to how our findings were aligned to the Walt & Gilson triangle. All findings were reviewed and refined prior to being measured against intended policy processes [23].

To ensure trustworthiness, the data were shared with some of the participants for the purpose of giving them an opportunity of clarifying the data. In addition, the third author re-coded the data, cross checked the entire analysis process and discrepancies were resolved, this ensured dependability of the findings [27]. The use of these different data collection tools enabled triangulation of the study findings.

## Results

**Fig. 2:**
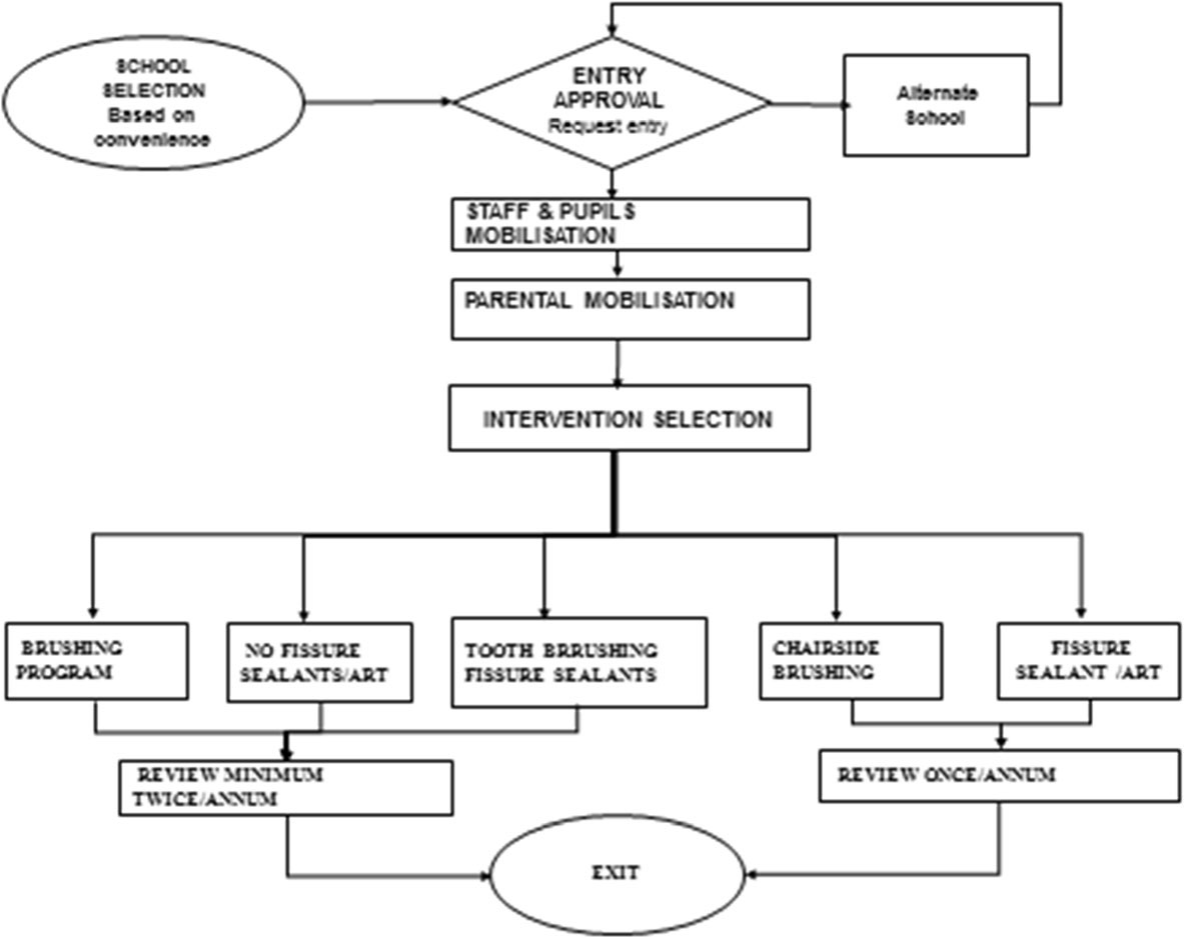
Map of actual implementation. This map was derived from the results obtained from the direct observations and process maps

The results illustrate the implementation of the programme using the broad themes of the policy analysis triangle [20]. Within the broad themes are sub-themes as displayed in Table 1. For content, the oral hygienist’s interpretation of the content and its delivery at the schools is outlined. The context illuminated structural environmental factors at play in the school settings and the influence of actors in the implementation process is discussed. In addition Fig. 2 is included to demonstrate the actual observed implementation, and two case observations in two contrasting schools are outlined. The final section then links in the Walt & Gilson summary triangle of the study findings.

**Table 1:** Summary table of themes and subthemes

Themes	Subthemes
***Processes of actual implementation***	School selection and entry
	Relational links
	Impacts of poor mobilization
***Perceptions of policy content***	Poor knowledge on policy content
	Uncertainty in delivering the content
	Dissatisfaction with programme content.
***Contextual factors affecting implementation***	Barriers to tooth brushing programme
	High service demands
	Inadequate infrastructure and equipment
	School food environment
***Actors influencing the implementation process***	Oral Hygienists
	Teachers
	Parents
	Dental assistants
	Snack vendors

### Processes of actual implementation

The implementation of actual activities as measured against proposed policies demonstrated that there was poor alignment in the implementation process as in (Figs. 1 & 2). First, the policy documents stipulate the need to carry out a community assessment before the selection of schools and initial entry. None of the oral hygienists used community assessments to inform school selection but rather relied on relational links.

*“ I have been coming to the school since 2006, I choose schools according to the cooperation of the school principal and staff” (Participant 1).*

This subsequently led to easy entry into the schools as all participants selections were based on historic working relationships.

*“I came at the beginning of the year to negotiate, but they are used to me so I was given entry” (Participant 3).*

Upon entry at a school, the oral hygienists were meant to mobilize school staff and learners in order to create oral health awareness and introduce the programme to the school community. Seven out of the ten study participants conducted this activity as exemplified below:

***“****At the beginning of the year, when I came to the school, I did education to everyone and demonstrated on the model” (Participant 1)*

*“I came to implement the brushing programme on January 17. First I trained the teachers and HOD (Head of Department) then went from class to class to educate the learners” (Participant 2)*

The few that did not mass mobilize opted to provide individualized education while the learners were in the dental chair. However they experienced significant challenges in obtaining support for the programmes. One participant cited:

*“Tooth brushing programme was rejected at the school. There were many challenges and teachers have no time [for the implementation process]” (Participant 3).*

Another participant concurred when she said:

*“Teachers don’t have time, I feel the programme should be implemented at grade R”(Participant 5).*

Furthermore, the lack of mobilization resulted in poor parental cooperation in terms of parents responding to the consent forms or referral letters. Parental cooperation was poor due to limited understanding of the programme and fear of cost implications that could arise if their children needed more care.

*“Some parents don’t sign consent for fear that they might need to pay for services.” (Participant 3).*

There were variations in terms of carrying out key activities as the oral hygienists focused on aspects of services that were feasible for them to carry out within their given circumstances (Fig. 2). One participant expressed her frustrations when she said:

*“You can’t do all programmes at the school, time doesn’t allow” (Participant 4).*

The factors outlined were a hindrance to creating a platform favourable for implementing the key processes.

### Perceptions of policy content

There was acknowledgement that the oral health policy was widely disseminated, yet the majority of participants had not been officially trained on the policy. Hence there was general uncertainty on how programme activities were to be delivered and how emerging challenges were to be addressed. They also reported on erratic policy changes that affected delivery of activities. Participants said:

*“You find that you start with old method then for next school you have to change” (Participant 1).*

*“Initially, we used to screen without consent, now the rules have changed. We are back to getting consent for the screening and then consent for treatment” (Participant 3)*

Consequently, rapid changes and inconsistencies in interpreting policy content resulted in oral hygienists using their discretion in implementing services.

*“Erratic policy changes have led to devising my own policy of screening only two grades in a year so as to finish in 2-3 weeks” (Participant 4).*

Challenges relating to the content of oral health policy, together with a lack of preparedness and insufficient funds allocated for the programmes led to most oral hygienists feeling that the programmes were not beneficial. Participants said:

*“I am not satisfied with the programme, the problem is with the department, money is tight, we sometimes have to rely on Colgate [ … ] Sometimes Colgate delivers late or numbers are not adequate for all the children” (Participant 1)*

*“I have been in the programme for years but I do not know if there is progress on not.” (Participant 6).*

Most challenges relating to the content of the policy were attributed to high level policy makers with poor knowledge of prevailing contextual challenges. One oral hygienists re-iterated:

*“Policy makers don’t pay attention to what is happening on the ground” (Participant 5).*

Content was therefore not well understood and appeared to have added no value to the implementation process.

### Contextual factors affecting implementation

Prevailing contextual challenges influenced the decisions on whether or not to carry out a tooth brushing programme. Five oral hygienists were not able to carry out the programme as the infrastructure at the school was inadequate; there was poor access to water, sanitation and a lack of wash basins. In addition the schools had large class numbers; hence they abandoned the brushing programme and instead focused on providing fissure sealants and ART to a few pupils who had parental consent for treatment. This led to completing a particular school sooner and covering more schools in a year.

*“Challenges include not enough basins, monitoring children was difficult, it would take two hours to do about 56 children in a class” (Participant 5).*

*“I try to cover as many schools in my area, so I am not likely to come back to the same school, same year” (Participant 7).*

The oral hygienists believed that their set targets were unattainable and unrealistic amidst the logistical challenges they experienced.

*“Policy is unreasonable, unrealistic and not feasible” (Participant 5).*

Other challenges that were cited included the lack of dental assistance, as this affected the ability to perform services that were technically sensitive such as fissure sealants and ART. In addition, there was defective equipment such as poor suction pressure in portable equipment. The following excerpts illustrate these findings:

*“Suction is not functioning, I have to improvise etch and children need to run to the tap to rinse” (Participant 7).*

Participants also reported that theft of tooth brushes and paste was very common and this hindered them from demonstrating brushing techniques. As a result, teachers would occasionally ask learners to store their brushes at home;

*“I was planning to continue with the brushing programme but the kids didn’t come with brushes today. Brushes are being stolen” (Participant 1.*

*“Tooth brush are getting lost, when you do a follow-up, then you need to be giving them more brushes” (Participant 2).*

Although most schools had government feeding initiatives which were nutritionally based, the school food environment was compromised as high caloric foods and sugar sweetened beverages were easily accessible from tuck shops and snack vendors at the schools. Moreover, a large number of the vendors were parents or community residents whose relationship with the school could not be put at stake:

*“There is a school nutrition programme which has two meals a day, morning and lunch breaks, yet tuck shops provides kotas and sweets, and many children also drink SSBs from the tuck shops” Participant 4.*

*“There are vendors selling sweets and chips on the ground, some of the vendors are school parents” Participant 1.*

This situation made health promotion difficult, and hence, most oral hygienists were not happy with the environment in which they worked. One participant said:

*“[I am] Not satisfied with the programme as I work in a compromised environment” (Participant 7).*

Due to such competing challenges, some oral hygienist felt that the programme had little impact on the oral health of pupils. One participant re-iterated:

*“I see no impact; I am just frustrated with the programme” (Participant 7).*

The oral hygienists had to contend with prevailing contextual circumstances that they had no control over. These challenges deterred the implementation of recommended programme activities.

### Actors influencing the implementation process

There were five groups of actors that directly or indirectly influenced the implementation of the programme in various ways; these included the oral hygienists, teachers, parents, dental assistants and snack vendors. Although the oral hygienists were well aware of the importance of the programme, there was general dissatisfaction with regards to how they were expected to carry it out. Hygienists were well aware that adequate support from the community at large was necessary for a successful programme. This was reflected by the statement:

*“The type of services I offer is determined by the level of cooperation or the accommodation I receive from the schools and also availability of facilities”* (*Participant 3)*.

Most of them resorted to improvising their techniques. For instance, the hygienists who were not able to offer coordinated brushing programmes resorted to getting each individual child to brush before being treated in the dental chair. This resulted in neglecting the children who were not scheduled to have treatment. The application of fissure sealants on teeth was also compromised as nearly all the saliva suctions were dysfunctional. Pupils were expected to rinse and spit at the nearest tap drain between treatments. This process prolonged the working time and compromised the retention of the placed fissure sealant.

Teachers played a key role in the supervision and motivation of learners in adhering to the oral hygienists programme. However this support was missing as the teachers had busy schedules and oral health activities were not prioritized.

*“Not all teachers adhere to the programme, they are non-cooperative” (Participant 5).*

Lack of cooperation from teachers led to some schools rejecting some activities in their schools as reported by one participant:

*“Tooth brushing programme was rejected at the school. There were many challenges and teachers have no time” (Participant 3).*

The parents were key actors when it came to consenting for the learners, especially those needing referrals for further treatment. However, more than half of the oral hygienists reported that most parents were not co-operative at following up on their children’s clinic referrals, welfare needs and signing consent forms for screening or referral.

*“Parents are uncooperative and relegate a lot of responsibilities to schools” (Participant 2).*

Dental assistants were important missing actors in the provision of oral health services at the schools. None of the oral hygienists were partnered with a dental assistant as there were staff shortages at the clinic facilities. This was a barrier to providing some of the technically sensitive procedures and hence some oral hygienists did not provide ART unless they had a dental assistant.

*“I only do ART if I have some assistant, but I actually prefer to do it (ART) if I am in the clinic in case I experience some complications”. Participant 10.*

The snack vendors were informal traders residing in the community, some schools allowed them inside the school premises, and at other schools they were outside the school grounds. This type of trading was permitted as it was a way of growing the local economy and allowing unemployed parents residing in the area to generate some income. The presence of these vendors counteracted the healthy eating patterns the oral hygienists are working to cultivate.

The actors were found to have a strong central role in the interplay between the elements of processes, content and context. The parents, teachers and snack vendors derailed the processes of implementation by not having an understanding of the content and its benefits. This was reflected by the lack of compliance in the referral system, poor support of the tooth brushing programme and provision of unhealthy snacks at the school sites. The oral hygienists unfortunately found themselves constrained by the other actors.

## Case observations

The following case observations below provide an illustration on how elements of processes, content, context and actors are intrinsically linked and furthermore affect policy implementation on a typical working day. The cases were selected from two participating schools that were experiencing distinct differences in terms of school environments and availability of resources.

### Case observation 1

Participant 3 worked in a remote area, which were high crime and poverty rates. The infrastructure of the school was noticeably poorly maintained, with paint peeling off the walls, dysfunctional toilets, makeshift reception desk and broken metal desks stacked up behind classrooms. A third of the buildings were portable cabins that were not climate controlled, and she was working in one of the rooms. Her operator chair had no backrest; she had no adequate light in the room and had no working dental suction. She was offering fissure sealants on the day of the visit. She received the children in the room in pairs, she placed pumice on toothbrushes requested the children to brush their teeth and go and rinse at the nearest tap in the school yard. When they returned, she administered etchant on one of the children, and sent them off to go and rinse again before she could place the fissure sealants and apply fluoride gel. When the first child was complete, they had to go back to class to call the next pair and the process was repeated. She took 30 min to undertake a procedure that takes 15 min, and hence she mentioned that she only gets to treat six children in the 4 hours at the school.

### Case observation 2

Participant 10 worked in an urban working class area. The school was evidently well managed and there were no apparent defects in school buildings that would affect learners’ health. There were strict security procedures at the main entrance to maintain school safety. A short glimpse of classrooms indicated that classes had few children and were controlled relatively well. The oral hygienist was working in a room purposed as a sick bay or medical room. She had well-functioning portable dental equipment that took about an hour to set up. She appeared relaxed and comfortable in her setting; she took her time in engaging with the children and making them comfortable. The teacher brought her four children at a time, and each child sat on the operator chair for the fissure sealants until their treatment was complete. She reported that she managed to complete about 10 children over the 4 hours at the school per day.

**Fig. 3:**
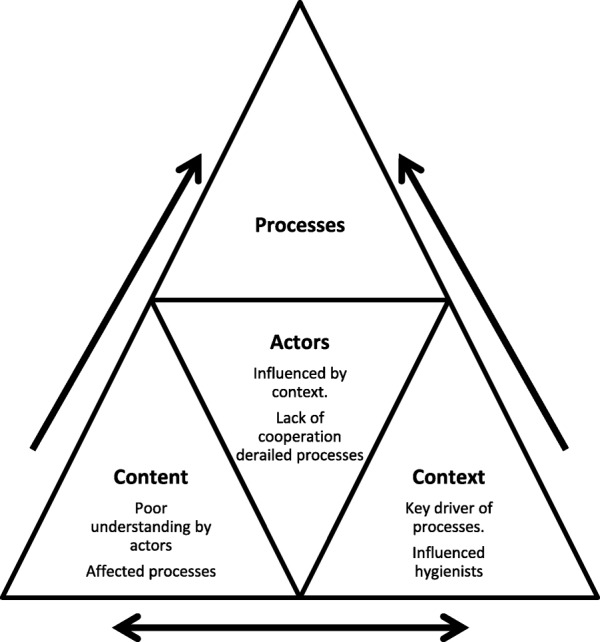
Summary triangle of the Walt & Gilson model

Figure 3, provides a summary on how the central role of actors derailed the processes of implementation by their poor understanding of the content and lack of co-operation. In turn the context in this case appeared as a strong driver of the processes of implementation. Furthermore, the contextual circumstances informed the type of content the oral hygienists were able to implement.

## Discussion

Our findings show that there was general misalignment between policy and practice, and that oral health programme delivery was not standard across the 10 schools. Policy processes were affected by poor prior planning, inadequate resources, poor school infrastructure and lack of support from key stakeholders. Furthermore, policy inconsistencies from management, coupled with the fact that the oral hygienists were not conversant with the policy hampered delivery of the policy content. The variations observed were often at the discretion of the oral hygienist in response to real contextual challenges which are common among South Africa’s frontline healthcare workers [28]. As a consequence, the oral hygienists traded policy directions with addressing practical challenges encountered.

It was clear that thorough assessment and planning did not occur before implementation as school selection and entry was not informed by the burden of need but rather on schools that were easily accessible. This was problematic as it disadvantaged the schools and children who may be in real need of services [1]. The processes of mobilization of teachers and learners had not been done such that the oral hygienists struggled to garner adequate support of teachers and parents for implementation of the programmes. This stems from the fact that there was no initial involvement of teachers and the wider community in the preliminary planning and entry into schools [6, 29].

Chamberlain et al. (2011) suggest that lack of exploration of needs and fit before implementation affects ongoing implementation processes [30]. This shortcoming was reflected by the insufficient consideration from the hygienists in terms of the level of resources and suitability of infrastructure available for them to carry out specific activities. Hence there were improvisations that affected the quality of service delivery, for instance providing fissure sealants without a functioning suction system. Such unplanned improvisations often resulted from poor co-ordination which has been associated with inadequate training of oral health staff [31]. Had the hygienists or their managers undertaken coordinated initial assessments, alternate feasible activities could have been planned beforehand [32].

The content of the policy was largely influenced by the oral hygienist’s perception of the policy and the context of the environment within which they were working [22]. Their understanding was vague and perceptions of policy implementation were not positive. They were uncertain of policy guidelines, and were frustrated with what was expected of them in the midst of challenges. Furthermore, policy changes were reported as erratic and resulted in hygienists being ill prepared to undertake the required tasks [33, 34]. Regular guidance and technical assistance could benefit the hygienists in such instances [32, 35].

Contextual challenges including limited resources and inadequate infrastructure informed decision making processes of how and what activities could be offered. This limited the number of children who could be covered for oral disease prevention. These complexities may be beyond the control of the hygienist as oral health budgetary allocations are generally inadequate in South Africa [31, 36]. Periodic monitoring and adaptations of the services by the Departments of Health and Basic Education could assist by regularly assessing for a good fit between services required and available resources to perform the required services [33].

There were high service demands in the midst of staff shortages and limited time to achieve set targets within the given school period. These challenges are echoed in similar health promoting school programmes across the African continent [32, 37, 38]. The teachers are often not able to assist the hygienists in coordinating the programmes as they have high workloads and large classes with which to contend. In addition they lack knowledge and confidence in oral health promotion [31]. The consequences of this high service demand resulted in an inability to revisit and review schools four times a year as recommended by policy. This minimal reinforcement may have hindered the programme from being fully accepted and adopted at the schools [39].

Although oral hygienists encouraged healthy eating, the schools had compromised healthy nutrition environments as snack vendors were allowed to become an integral part of the school. This has been a problem at South African public schools despite the National School Nutrition Programme being initiated in 2004 [31, 40]. Moodley et al. al (2015) attributes this challenge to the lack of mandatory regulation of food and beverages sold around school premises. The street vendors have been approached with suggestions to sell healthy snacks, however they claim that fruit is expensive, perishable and cannot be stored for long periods, hence it is more financially viable for them to sell processed snacks [31, 41]. Co-ordinated engagement processes between schools and community snack vendors are necessary to derive joint health promotion solutions that will benefit both pupils and vendors [42].

As actors, the oral hygienists were generally dissatisfied with the programme as they had to drive the processes of implementation in the midst of obstacles. However they attempted to optimize their services despite their circumstances [22]. The hidden actors such as the dental assistants and snack vendor had no direct involvement in the programme and yet influenced it significantly in terms of outcomes. The lack of dental assistants impacted on the hygienist’s ability to carry out certain activities and inhibited quality of care [43]. In turn, the presence of the snack vendors hindered the children from practicing healthy eating habits and protecting their oral health [31]. In this particular case it appeared that the actors are highly dependent on one another for successful implementation and therefore more effort should be placed on educating stakeholders on the content, value of the programme and collaborative support [44].

The above discussion has demonstrated how intrinsically linked the elements of processes, content, context and actors are in analyzing policy implementation [20]. In our study the actors and context influenced the processes of implementation and the delivery of the policy content. In turn the limited understanding and interpretation of the policy content by the actors influenced the processes of implementation.

The implications of these findings are that the policy recommendations were not feasible given the presenting contextual obstacles. In this case, regular monitoring, evaluation and root cause analyses could be needed to identify contextually relevant modifications and address areas of concern in the programme. In addition, a multidisciplinary effort in the planning and execution of such complex programmes is necessary for tackling the factors affecting oral health delivery at schools.

## Limitations

The school services were not observed over multiple visits and hence any change of patterns or trends in the services offered could not be reported on. However, multiple data sources including observations, interviews and policy documents were used to ensure that the phenomenon was explored from a variety of perspectives. In addition, during analysis, all the data were converged to ensure holistic understanding [23, 45].

## Conclusion

There was policy and practice misalignment and variations in the processes of implementation of oral health services across the 10 schools. This observation was partly the result of poor planning, inadequate understanding of policies, insufficient resources, poor school infrastructure and a lack of support from key stakeholders. The delivery of the programme was largely driven by contextual circumstances and poor knowledge of the policy content. Furthermore the context informed the degree of the content that could be delivered. In all, the interplay between the elements of the policy triangle illuminated the factors that were responsible for the extent of implementation of school oral health policy and programme.

## Supplementary information

**Additional file 1.** *The policy implementation gap of school oral health programmes in Tshwane, South Africa: A qualitative case study. *Interview guide.

## Data Availability

The datasets used and/or analysed during the current study are available from the corresponding author on reasonable request.

## Abbreviations

ART: Atraumatic Restorative Technique
SSB: Sugar Sweetened Beverages
HOD: Head of Department
Grade R: Reception Grade

## References

[1] Bourgeois DM, Llodra JC (2014). Global burden of dental condition among children in nine countries participating in an international oral health promotion programme, 2012–2013. Int Dent J.

[2] Kwan SY, Petersen PE, Pine CM (2005). Health-promoting schools: an opportunity for oral health promotion. Bull World Health Organ.

[3] Sohn W, Burt BA, Sowers MR (2006). Carbonated soft drinks and dental caries in the primary dentition. J Dent Res.

[4] Kassebaum NJ, Bernabé E, Dahiya M (2015). Global burden of untreated caries: a systematic review and metaregression. J Dent Res.

[5] National Department of Health South African National Oral Health Strategy. Pretoria: DoH; Available In https://www.health.goz.za/strategic/130-sd2005South-African-national-oral-health-strategy. 2010; Accessed 25 Sept 2018.

[6] Jürgensen N, Petersen PE (2013). Promoting oral health of children through schools–results from a WHO global survey 2012. Community Dent Health.

[7] Petersen PE, Kwan S (2004). Evaluation of community-based oral health promotion and oral disease prevention-WHO recommendations for improved evidence in public health practice. Community Dent Health.

[8] Statistics South Africa (2014). General Household Survey 2013. Retrieved from South Africa.

[9] United Nations Educational, Scientific & Cultural Organisation. Transforming education: The power of ICT policies. France: Unesco; 2011. Retrieved from unesdoc.unesco.org/ark:/48223/pf0000211842.

[10] Blumenshine SL, Vann WF, Gizlice Z (2008). Children's school performance: impact of general and oral health. J Public Health Dentistr.

[11] Garg N, Anandakrishna L, Chandra P (2012). (2012). Is there an association between oral health status and school performance? A preliminary study. Int J Clin Pediatr Dentistr.

[12] Van Wyk PJ, Van Wyk C (2004). Oral health in South Africa. Int Dent J.

[13] Thekiso M, Yengopal V, Rudolph MJ (2012). (2012). Caries status among children in the west Rand District of Gauteng Province, South Africa. SADJ.

[14] Reddy M, Singh S (2015). (2015a). Dental caries status in six-year-old children at health promoting schools in KwaZulu-Natal, South Africa. S Afr Dent J.

[15] Department of Health & Basic Education. Integrated School Health Policy. South Africa: National Department of Health and Basic Education; 2012. Avalable in https://serve.mg.co.za/content/2017/integratedschoolhealthpolicydbeanddoh.pdf. Accessed 02 May 2018.

[16] Department of Health, South Africa (2011). School Health Policy & Implementation Guidelines.

[17] Singh S, Myburgh NG, Lalloo R (2010). Policy analysis of oral health promotion in South Africa. Glob Health Promot.

[18] Matsoso MP, Fryatt R (2013). National Health Insurance: the first 18 months. SAMJ.

[19] National Department of Health (2015). National Health Insurance for South Africa: Towards Universal Health Coverage.

[20] Walt G, Gilson L (1994). Reforming the health sector in developing countries: the central role of policy analysis. Health Policy Plan.

[21] Faraji O, Etemad K, Sari AA (2015). Policies and programs for prevention and control of diabetes in Iran: a document analysis. Global J Health Sci.

[22] Lehmann U (2016). Understanding and Analysing Health Policy.

[23] Baxter P, Jack S (2008). Qualitative case study methodology: study design and implementation for novice researchers. Qual Rep.

[24] Wilgis M, McConnell J (2008). Concept mapping: an educational strategy to improve graduate nurses’ critical thinking skills during a hospital orientation program. J Contin Educ Nurs.

[25] Biggs JS, Farrell L, Lawrence G (2014). Applying process mapping and analysis as a quality improvement strategy to increase the adoption of fruit, vegetable, and water breaks in Australian primary schools. Health Promot Pract.

[26] Srivastava A, Thomson SB (2009). Framework analysis: a qualitative methodology for applied policy research.

[27] Bryman A (2001). Qualitative data analysis Social Research Methods.

[28] Walker L, Gilson L (2004). ‘We are bitter but we are satisfied’: nurses as street-level bureaucrats in South Africa. Soc Sci Med.

[29] Casamassimo PS, Lee JY, Marazita ML (2014). Improving children’s oral health: an interdisciplinary research framework. J Dent Res.

[30] Chamberlain P, Brown CH, Saldana L (2011). Observational measure of implementation progress in community based settings: the stages of implementation completion (SIC). Implement Sci.

[31] Reddy M, Singh S (2017). The promotion of oral health in health-promoting schools in KwaZulu-Natal Province, South Africa. S Afr J Child Health.

[32] Durlak JA, DuPre EP (2008). Implementation matters: a review of research on the influence of implementation on program outcomes and the factors affecting implementation. Am J Community Psychol.

[33] Dusenbury L, Brannigan R, Falco M (2003). A review of research on fidelity of implementation: implications for drug abuse prevention in school settings. Health Educ Res.

[34] Shea CM, Jacobs SR, Esserman DA (2014). Organizational readiness for implementing change: a psychometric assessment of a new measure. Implement Sci.

[35] Darlington EJ, Violon N, Jourdan D (2018). Implementation of health promotion programmes in schools: an approach to understand the influence of contextual factors on the process. BMC Public Health.

[36] Reddy M, Singh S (2015). Viability in delivering oral health promotion activities within the health promoting schools initiative in KwaZulu-Natal. S Afr J Child Health.

[37] Macnab AJ, Rozmus J, Benton D (2008). 3-year results of a collaborative school-based oral health program in a remote first nations community. Rural Remote Health.

[38] Lawal FB, Taiwo JO (2014). An audit of school oral health education program in a developing country. J Int Soc Prev Community Dent.

[39] Banfield M, McGorm K, Sargent G (2015). Health promotion in schools: a multi-method evaluation of an Australian school youth health nurse program. BMC Nurs.

[40] Faber M, Laubscher R, Laurie S (2013). Availability of, access to and consumption of fruits and vegetables in a peri-urban area in KwaZulu-Natal, South Africa. Matern Child Nutr.

[41] Moodley G, Christofides N, Norris SA (2015). (2015). Obesogenic Environments: Access to and Advertising of Sugar-Sweetened Beverages in Soweto, South Africa, 2013. Prev Chronic Dis.

[42] Lewallen TC, Hunt H, Potts-Datema W (2015). The whole school, whole community, whole child model: a new approach for improving educational attainment and healthy development for students. J Sch Health.

[43] Potgieter C, Naidoo S (2017). How effective are resin-based sealants in preventing caries when placed under field conditions*?*. S Afr Dent J.

[44] Gill W, Gill W (1994). Implementation: do those who implement decide?. Health Policy: An Introduction to Process & Power.

[45] Hussein A (2009). The use of triangulation in social sciences research: can qualitative and quantitative methods be combined. J Comp Soc Work.

