# Supplementary material for: The policy implementation gap of school oral health programmes in Tshwane, South Africa: a qualitative case study
Source: BMC Health Serv Res. 2020 Apr 21;20:338. doi: 10.1186/s12913-020-05122-8 (PMC7171775; doi:10.1186/s12913-020-05122-8)
Supplement: Supplementary file 1 — Additional file 1. *The policy implementation gap of school oral health programmes in Tshwane, South Africa: A qualitative case study. *Interview guide. [file 12913_2020_5122_MOESM1_ESM.docx]

**Additional File 1**

Interview guide for study: **The policy implementation gap of school oral health programmes in Tshwane, South Africa: A qualitative case study.**

The guide is informed by: WHO Information Series on School Health (2005); NDoH (2010).

**1. Demographic information**

- Location
- Position

**2. School health service**

- Describe the types of oral health services provided?
- Do your activities occur simultaneously?
- How do oral hygienists determine which activities happen at specific schools?
- Probes: what? why? how? threats &opportunities?

**3. School health education**

- Do you see oral health integrated into the school curriculum?
- How are the learning activities implemented?
- Do you see these activities as effective in promoting oral health and healthy behaviours and, if so, to what extent?
- What type of training is provided and to whom?
- What do teachers and others think of the curriculum?

**4. Policy**

- Does the school have a comprehensive oral health policy or, if not, policies that relate to oral health?
- Is/are the policy or policies implemented and enforced as written?
- Are resources and responsible people designated to support oral health promotion inventions?
- How are the students, parents, school staff and members of the community involved in the planning, development and implementation of policies?

**5. Goals and objectives**

- How are these programmes meeting the goals and objectives that have been set by government?
- In your view do you think these programmes are working?
- What would you like to see that would convince you that the programmes are working?

**6. Health promotion for school staff**

- Are there any tailor-made oral health promotion programmes for staff?
- If yes, what do the staff members think of them?
- Would you say they have they been effective and how?

**7. School and community relationships and collaboration**

- Do you know if there is community involvement of these programmes at schools?
- To what extent is the community involved?
- Does the school provide any oral health training courses for parents and members of the community?
- If so, what type of training has been available and do you think it is effective?

**8. Nutrition and food services**

- How well is oral health integrated into the healthy nutrition interventions in school and the community?
- How are the food service providers involved in promoting oral health? Are they aware of their role?
